# Effects of different training intensities in high-intensity interval training (HIIT) on maximal aerobic velocity, hematological and muscle-damage markers in healthy young adults

**DOI:** 10.1186/s13102-022-00550-x

**Published:** 2022-08-22

**Authors:** Fatma Rhibi, Abderraouf Ben Abderrahman, Jacques Prioux, Cain C. T. Clark, Benoît Bideau, Sophia Besbes, Anthony C. Hackney, Urs Granacher, Hassane Zouhal

**Affiliations:** 1 Laboratory of Biomonitoring of the Environment, Faculty of Science of Bizerte, University of Carthage, Bizerte, Tunisia; 2 Movement, Sport, Health and Sciences Laboratory (M2S), UFR-STAPS, University of Rennes 2-ENS Cachan, Av. Charles Tillon, 35044 Rennes Cedex, France; 3 High Institute of Sport and Physical Education, Ksar-Saïd, Manouba University, Tunis, Tunisia; 4 Centre for Intelligent Healthcare, Coventry University, Coventry, CV1 5FB UK; 5 Biochemical Laboratory, Hospital of Kassab, La Manouba, Tunis, Tunisia; 6 Department of Exercise and Sport Science, Department of Nutrition, University of North Carolina, Chapel Hill, NC USA; 7 Department of Sport and Sport Science, Exercise and Human Movement Science, University of Freiburg, Sandfangweg 4, 79102 Freiburg, Germany; 8 Insitut International Des Sciences du Sport (2I2S), 35850 Irodouer, France

**Keywords:** Performances, Immunological parameters, Intermittent exercise, Hematological parameters

## Abstract

This study aimed to examine the effects of two high-intensity interval training programs (HIIT) on maximal aerobic velocity (MAV), hematological variations and muscle damage markers in young healthy adults. Twenty-nine male physical education students, aged 20.3 ± 3.3 years, volunteered to participate in this study, and were randomly assigned to a control group (CG, n = 9) or two intervention groups (group 1 or 2). Intervention group 1 (n = 10) exercised at 100% of their MAV (EG_100_) while group 2 (n = 10) exercised at 110% MAV (EG_110_). Before and after the eight week training program, blood samples were drawn at rest, before, and after an intermittent exercise. Aspartate aminotransferase (ASAT), alanine aminotransferase (ALAT), C reactive protein (CRP), creatine kinase (CK) concentrations and hematological parameters (white blood cells [WBC], monocytes [MO], lymphocytes [LY], neutrophil [NE]) and lactate dehydrogenase (LDH) were measured. Post-hoc tests showed that MAV was significantly higher in EG_110_ compared to EG_100_ after HIIT (*p* < 0.01, η_p_^2^ = 0.05), whilst ALAT, ASAT, and CPR were significantly lower (*p* < 0.01; 0.02 < η_p_^2^ < 0.11) in EG_110_ compared to EG_100_. Moreover, post-hoc tests indicated that LY decreased significantly (*p* < 0.001, η_p_^2^ = 0.21) only for EG_110_. Furthermore, there were significant positive correlations for both EG_100_ and EG_110_ between MAV and ALAT (r = 0.66, *p* = 0.044 and r = 0.64, *p* = 0.041 respectively), CK (r = 0.67, *p* = 0.031 and r = 0.86, *p* = 0.030, respectively), LDH (r = 0.74, *p* = 0.014, and r = 0.071, *p* = 0.021, respectively). In addition, there was a significant positive correlation for both, EG_100_ and EG_110_ between MAV and LY (r = 0.79, *p* < 0.01; r = 0.72, *p* < 0.05, respectively). Concerning the relationship between MAV and NE, there was a significant positive correlation (r = 0.66; *p* < 0.05) only for EG_110_. Findings from this study revealed that HIIT at 110% MAV was more efficient to improve MAV and reduce muscle damage. In addition, we observed significant associations between performance improvements (MAV) and markers of muscle damage.

## Background

It is well-established that acute high intensity physical exercise can have negative effects on inflammatory mediators [1, 2]; concurrently, however, long-term physical training has the potential to enhance performance markers such as maximal aerobic velocity (MAV), improve immune function, and reduce inflammation [3]. Training intensity appears to play a key mediating role in directing the effects of exercise bouts towards biopositive or negative responses. In fact, several studies showed signicant MAV improvements after 3.5 to 7.0 weeks of HIIT [4–6]. Recently, Rhibi et al. [5] showed a significant MAV enhancement after 8 weeks of HIIT in male physical education students. Furthermore, the exercise immunology literature is consistent regarding post-exercise cell count increases (leucocytosis, neutrophil [NE], monocytosis [MO], and lymphocytosis [LY]) after a single bout of high-intensity swimming [6, 7] or cross‐country skiing [8]. Brancaccio et al. [9] showed that white blood cells (WBC) increased significantly during high-intensity maximal tasks. There is evidence that physical activity may also alter homocysteine metabolism by increasing protein and/or methyl-group turnover [10]. In fact, Nieman [11] proposed a model of immune response to training named the inverted-U model. In fact, the immune status was gradually improved in response to an increase in physical activity. However, it was decreased in response to high intensity training. Moreover, different studies showed a slight decrease in the immune parameters in response to high intensity training or overtraining programs in athletes [12, 13]. However, no modification was observed in mitogens activity after 60 min of running at 95% of VMA in trained runners. Interestingly, there is also evidence that the leucocytosis induced by intense exercise (90–105% of maximal aerobic velocity [MAV]) could be due to the general increase in muscle damage [14].

It is well-established that prolonged physical exercise results in transient elevations of biochemical markers of muscular damage such as aspartate aminotransferase (AST), alanine aminotransferase (ALT), creatine kinase (CK), and lactate deshydrogenase (LDH) [9]. Previous studies reported a signifcant increase in CRP [15, 16], CK [15–17], and LDH [15, 18] after training programs. In fact, Anđelković et al. [15] found that CRP, CK, and LDH concentrations increased after 90 days of soccer training. Silva et al. [16] showed an increase in CRP and CK levels at the end of a soccer season in professional soccer players. In contrast, Requena et al. [19] observed that CK remained unaltered and LDH concentration decreased after the off-season rest period. Gharahdaghi et al. [20] showed a significant reductions in LDH and CK concentration at rest in response to 4 weeks of high-intensity aerobic training.

The literature also suggests that long-term training could affect the different muscle damage markers mentioned above [21]. Thus, it is important to determine the optimal intensity and duration during high-intensity interval training (HIIT) to achieve performance improvements (e.g., MAV) and at the same time to minimize the muscular damage and the potential injury risks [22]. Likewise, exercise intensity is an important factor contributing to sarcolemma disruption, allowing the release of enzymes of damaged muscle into the blood [23]. In recent years, trainers/coaches and athletes have sought contemporary training modes to yield performance improvements and induce optimal adaptation [24]. Indeed, moderate exercise (55–75% MAV) stimulates the immune system and may be somewhat responsible for exercise related reduction in fatigue and illness [25]. However, high intensity exercise (90–100% MAV) induces immunosuppression in the recovery period and may explain the increased risk of infection in athletes [25]. In endurance sports, during the cycles of high training volume and intensity that include consecutive training sessions with little recovery time in between, athletes may experience a temporarily diminished performance concomitant with an immunodepression state [26, 27]. Indeed, stiffness and soreness following a period of exercise are less when the exercise is repeated a week later; the result of adaptation by the muscle [28]. However, none of these studies have examined the variation in muscular damage markers in response to a HIIT exercise program, despite the known efficiency of this training type for performance improvement.

In addition, HIIT protocols enable individuals to maintain sub-maximal (90% MAV at least), maximal (100% MAV), or supra maximal (> 100% MAV) intensities. In this context, Millet et al. [29] showed that the time recorded at 90% of V̇O_2max_ were significantly higher during 30 s IE at 105% V̇O_2max_ than IE at 100% V̇O_2max_. Ballor and Volovsek [30] showed that increasing exercise intensity (from 90 to 110% MAV) could improve the mean V̇O_2_ (from 63.5 to 69.6% MAV) measured during IE. In addition, Thevenet et al. [31] showed that no significant difference was found in t90 V̇O_2max_ or t95 V̇O_2max_ values in response to 100% or 110% MAV IE. Indeed, IE at 100–110% MAV did not allow all participants reaching at least 90 or 95% of V̇O_2max_ (only 2 out of 9 tested participants) [31]. Thus, it is plausible that higher exercise intensities might allow a greater improvement in aerobic performance and the fatigue resistance at high intensity effort. However, a high exercise intensity can induce central or peripheral fatigue which could possibly impair sports performance [32]. To our knowledge, there are no published longitudinal data pertaining to the effects of increasing the exercise intensity of intermittent exercise and the resultant muscle damage and hematological responses. Given the lack of research in this area, the aim of the current study was to examine the influence of eight weeks of HIIT, at different intensities (100% MAV vs. 110% MAV), on MAV, muscle damage markers and hematological parameters in young adults. Based on the extant literature [33, 34], we hypothesized that increasing training intensity could lead to better muscular adaptations in order to reduce muscular damage markers after short and intense intermittent exercise, and that any HIIT would yield greater changes than the control group.

## Methods

### Participants

Twenty-nine male physical education students volunteered to participate in this study, and were randomly assigned into three groups: a control group (CG, n = 9; age: 22.0 ± 1.2 years) and two experimental groups (EG). EG_100_ comprised n = 10 individuals aged 21.4 ± 1.1 years and EG_110_ included n = 10 participants aged 21.9 ± 1.3 years. More information on the experimental groups will be provided in the next paragraph. Age of participants and their physical characteristics were measured before and after HIIT and are displayed in Table 1. The participants were informed of all the procedures of the experiment including performance of the exercise training protocols and possible risks associated with its administration in accordance with the Helsinki Declaration. Prior to participation, the participants underwent a medical examination and were fully informed about the experimental procedures and signed consent was obtained from the participants. Approval was granted by the Research Ethics Committee of the University of Rennes 2, Rennes, France.

**Table 1:** Mean (± SD) values for age and anthropometric characteristics for all groups

Variables	Group	Pre	Post	*p* (eta-squared [η_p_^2^])		
				main effect of group	main effect of time	group x time interaction
Body mass (kg)	EG_100_	72.7 ± 8.1	72.2 ± 7.7	0.532 (0.12)	0.910 (0.01)	0.233 (0.13)
	EG_110_	72.9 ± 6.2	72.4 ± 6.1			
	CG	72.5 ± 4.2	72.3 ± 3.7			
Body height (cm)	EG_100_	178.8 ± 5.4	179.0 ± 5.3	0.786 (0.03)	0.340 (0.17)	0.345 (0.08)
	EG_110_	180.9 ± 6.8	181.0 ± 6.8			
	CG	180.1 ± 6.2	179.9 ± 5.7			
BMI (kg.m^−2^)	EG_100_	22.7 ± 1.3	22.5 ± 1.2	0.368 (0.09)	0.162 (0.12)	0.197 (0.11)
	EG_110_	22.3 ± 1.4	22.1 ± 1.3			
	CG	22.4 ± 1.0	22.3 ± 0.9			
BF (%)	EG_100_	11.6 ± 1.1	11.5 ± 1.2	0.791 (0.02)	0.103 (0.16)	0.174 (0.13)
	EG_110_	11.4 ± 1.5	11.3 ± 1.4			
	CG	11.8 ± 1.4	11.9 ± 1.4			
MAV (km.h^−1^)	EG_100_	15.82 ± 1.61	16.74 ± 1.48 a	0.591 (0.05)	< 0.001 (0.71)	< 0.001 (0.61)
	EG_110_	16.14 ± 2.10	17.58 ± 2.16 ac			
	CG	16.09 ± 1.95	16.13 ± 1.83			

Data are mean values (± SD), BM, body mass; BMI, body mass index; BF, Body fat; EG_100_, trained group with 100% MAV (n = 10); MAV, Maximal aerobic velocity; EG_110_, trained group with 110% MAV (n = 10); CG, control group (n = 9); HIIT, high intensity interval training program; pre-test, before HIIT; post-test, after HIIT

a: significant difference between before and after HIIT

c: significant difference between EG_100_ and EG_110_

### Experimental approach

All subjects visited the laboratory for a familiarization session to get to know the applied experimental set-up (e.g., tests). During this session, anthropometrics (i.e., body height and mass, percentage body fat) were taken. All measurements were performed by the same investigator in accordance with the positions and techniques established by the International Biological Program [35].

Study exclusion criteria comprised any contraindications to maximal exercise testing (e.g., cardiovascular or pulmonary disease), endocrine disorders, and metabolic syndrome symptoms (e.g., hypertension). All participants were healthy, without any injuries during the past six months prior to the start of the study.

**Fig. 1:**
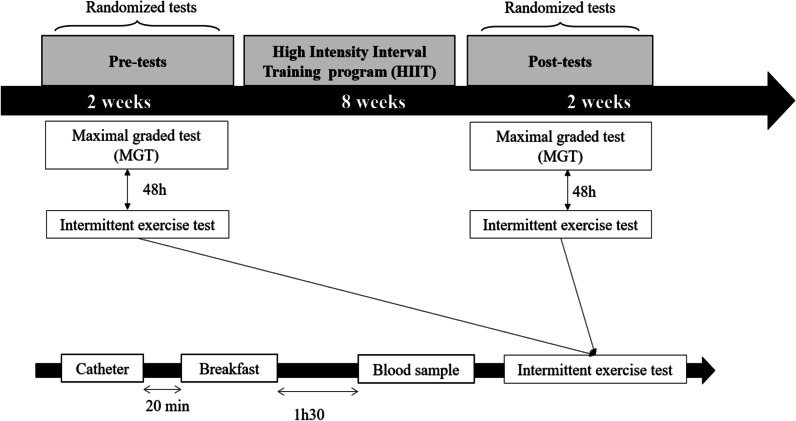
Study design for both experimental groups and control groups and experimental design for blood sampling

Before and after HIIT, all participants performed a maximal graded test [36] to determine their maximal aerobic velocity (MAV). Thereafter, subjects in the EG_100_ and EG_110_ groups carried out an intermittent exercise test consisting of repeated 30 s intensive runs at 100% MAV (EG_100_) or 110% MAV (EG_110_) (Fig. 1). Between the bouts, a 30 s active recovery was allowed (50% MAV). The runs were repeated until exhaustion. The CG only performed the maximal graded test and the intermittent exercise test before and after HIIT. Participants of both EG_100_ and EG_110_ performed another maximal graded test at the mid of the HIIT program (at the 5^th^ week) in order to assess MAV and to update the training speeds for the remaining weeks of the training program. All tests took place in the morning and were performed until full exhaustion on a 400 m outdoor tartan track, at the same time of day, with 48 h of rest between tests. Our study took place in ambient temperatures ranging from 16 to 22 °C and humidity from 50 to 75% with a wind speed < 2 m/s. For each test, the participants were verbally encouraged to run at maximal effort. Testing was stopped if participants could not maintain the required speed or if they stopped the tests due to exhaustion. Before and after the HIIT programs, all tests (maximal graded test and intermittent exercise) were completed within 2 weeks (Fig. 1).

### Anthropometric measurements

Body mass was measured to the nearest 0.1 kg, with the participants in light clothing and unshod, using an electronic scale (Kern, MFB 150K100). Height was determined to the nearest 0.5 cm with a measuring tape fixed to the wall. All measurements were performed by the same examiner, in accordance with the positions and techniques established by the International Biological Program. Percent body fat was determined using four skinfolds and a Harpenden caliper [37]. The fat free mass was calculated by subtracting the fat mass from the body mass.

### Maximal graded test

To determine participants’ MAV, a maximal graded test was performed on a track, in a stadium according to Cazzorla and Léger [36] The initial speed was 8 km h^–1^ and this was increased by 0.5 km h^–1^ every minute. The running pace was given by an examiner, equipped with a whistle and a chronometer and made a short sound when the subject had to pass a cone to be able to maintain a constant speed. A longer sound marked the change in the running stage. The velocity at the finally completed stage was determined as MAV. The accuracy of MAV was considered to be equal to the velocity during the previous stage plus 0.5 km h^−1^ [38].

### Intermittent exercise (IE) test

Participants performed, as long as possible, a 30 s run at 100% MAV (EG_100_) or 110% MAV (EG_110_) alternating with 30 s active recovery (50% MAV). These tests were carried out on the same track as the maximal graded test. Before each test, we calculated, as a function of the MAV of each subject, the required distance to be covered during the 30 s intensive runs at 100% MAV, 110% MAV, and the 30 s active recovery at 50% MAV. During the recovery period, a longer sound was made at mid-period (15 s) to inform the athletes of the remaining time for the end of recovery and make a U-turn. The distance performed for 30 s at both intensities (100% or 110% MAV) was calculated based on the following equation: Distance = Velocity × time. The 30 s IE was preceded by a standardized warm-up consisting of 10 min continuous jogging, followed by 5 min of the participant’s usual stretching routine, five short bursts of accelerations on the track, and 2 min rest.

### High intensity interval training programs

EG_100_ and EG_110_ groups participated in the HIIT program three times per week for a total of 8 weeks making up 24 exercise sessions (Table 2). The HIIT sessions were separated by at least 48 h to allow sufficient recovery. All HIIT sessions included three different periods according to the procedures previously described by Rhibi et al. [5, 39]. In brief, the sessions started with a standardized warm-up which consisted of 15 min continuous jogging, followed by 5 min dynamic stretching exercises and 5 short bursts of accelerations on the track. During every training session on the track, there was one subject per lane. All different distances for each athlete (running and recovery intervals) were fixed by the examiner before every exercise session. The subjects began from a standing position, behind a cone. Thereafter, they performed the HIIT session. For these training sessions, the subjects’ pace was provided by an examiner emitting sounds at regular intervals up to the end of the exercise session. During the 30 s recovery period, subjects had to cover a distance determined according to their own MAV. At the end of each HIIT session, subjects cooled down for ~ 5 min by running at low intensity, and performing dynamic stretching. Two members of our laboratory supervised all HIIT sessions. The two intervention groups (EG_100_ and EG_110_) performed similar training volumes across the study period. In contrast to EG_100_ and EG_110_, CG did not participate in any additional exercise program, but continued their regular compulsory physical education program at the university.

**Table 2:** Exercise characteristics of the two intervention groups

Week	Week 1	Week 2	Week 3	Week 4	Week 5	Week 6	Week 7	Week 8
*EG*_*100*_								
Set × (repetition)	2 × (8 × 30sIE)	2 × (9 × 30sIE)	2 × (10 × 30sIE)	2 × (11 × 30sIE)	2 × (10 × 30sIE)	2 × (12 × 30sIE)	2 × (12 × 30sIE)	2 × (11 × 30sIE)
Intensity	100% MAV/50%	100% MAV/50%	100% MAV/50%	100% MAV/50%	100% MAV/50%	100% MAV/50%	100% MAV/50%	100% MAV/50%
TL	1200 ATU	1350 ATU	1500 ATU	1650 ATU	1500 ATU	1800 ATU	1800 ATU	1650 ATU
*EG*_*110*_								
Set × (repetition)	2 × (8 × 30sIE)	2 × (9 × 30sIE)	2 × (10 × 30sIE)	2 × (11 × 30sIE)	2 × (10 × 30sIE)	2 × (12 × 30sIE)	2 × (12 × 30sIE)	2 × (11 × 30sIE)
Intensity	110% MAV/50%	110% MAV/50%	110% MAV/50%	110% MAV/50%	110% MAV/50%	110% MAV/50%	110% MAV/50%	110% MAV/50%
TL	1280 ATU	1440 ATU	1600 ATU	1760 ATU	1600 ATU	1920 ATU	1920 ATU	1760 ATU

MAV, Maximal aerobic velocity; EG_100_, 100% MAV training group (n = 10); EG_110_, 110% MAV training group (n = 10); TL, Training load; ATU, Arbitrary training units; Example: [2 × (8 × 30 s / 30 s) 100/50% MAV]; It means that the participant had to run two series with eight repetitions each 30 s composed of 30 s running at 100% of MAV and 30 s active recovery at 50% of MAV. The participant recovers passively 5 min between each series. Each session was repeated 3 times per week

### Blood sampling and analysis

Pre and post the intervention period, blood samples were taken before the 30 s intermittent exercise test. All venous blood samples were drawn upon arriving at the laboratory, where a heparinized catheter (Insyte-W, 1.1 mmo.d. × 30 mm) was inserted into an antecubital vein. Subjects then rested quietly, for 20 min. Thereafter, a blood sample (10 mL) was taken to determine resting concentration. Samples were placed in an ice bath and centrifuged immediately. Aliquots of the resulting plasma were stored at − 80 °C until analyzed. Blood was collected in two tubes: the first heparinized tube was used to determine aspartate aminotransferase (ASAT), alanine aminotransferase (ALAT), C reactive protein (CRP), creatine kinase (CK) and lactate dehydrogenase (LDH) concentrations. The second EDTA tube was used to determine hematological parameters (i.e., white blood cells—WBC) and principal derivative subpopulations such as monocytes (MO), lymphocytes (LY) and neutrophil (NE). The ALAT, ASAT, CRP, CK and LDH concentrations were determined using a multiparametric analyzer Konelab 30™ (Thermo Electron Corporation). ALAT and ASAT activities were determined enzymatic method using kinetic method (UV). The intra-assay coefficient of variation for the ALAT and ASAT kit was 1.4 and 1.8%, respectively. CRP activity was determined using an Immunoturbidimetry method. The intra-assay coefficient of variation for the CRP kit was 1.7%. CK activity was determined using UV method (IFCC) by the N-acetyl-cysteine method. The intra-assay coefficient of variation for the CK kit was 1.8%. LDH activity was determined by enzymatic rate method (IFCC). The intra-assay coefficient of variation for the LDH kit was 1.1%. Haematological parameters were generally performed within 3 h in a multichannel automated Hematology Analyzer Sysmex XS-1000i. We measured simultaneously red blood cells, WBC and principal derivative subpopulations such as NE, LY and MO.

### Statistical analyses

Data were reported as means and standard deviations (± SD). Normality of data was tested and confirmed for all variables using the Kolmogorov–Smirnov procedure. Levene’s test was used to determine homogeneity of variance. Test–retest reliability for all tests was assessed using intra-class correlation coefficients (ICCs) [40]. The effects of exercise were evaluated using a two-way analysis of variance (ANOVA) with repeated measures (3 groups × 2 times). If group by time interactions turned out to be significant, Bonferroni adjusted post-hoc tests were computed. Greenhouse–Geisser corrections were used when the assumption of sphericity (Mauchly’s test) was violated. Partial eta-squared (ηp^2^) was calculated to assess the practical significance for all ANOVA outcomes. Values of 0.01–0.059, 0.06–0.13, and ≥ 0.14 were considered as small, intermediate and large effects, respectively [41]. We also calculated predefined contrasts analyses [42] to specifically test the following hypothesis, H1) any HIIT condition would yield greater improvements in the outcome measures than the control group. Accordingly, we compared the control condition vs EG_100_ and EG_110_ (coded as − 0.667, 0.333, and 0.333, respectively). This approach yielded a comparison of one (or more) condition(s) vs. the grand mean of the specified contrasts. Indeed, post-hoc analysis, while useful, does not, alone, yield sufficient insight into multiple levels or detailing patterns in response; whereas, contrast analysis allows researchers to test theory-driven expectations directly against empirically derived group or cells means [43, 44]. In addition, we plotted pre-post changes (deltas Δ) of performance measures (MAV) with deltas in hematological variations and muscle damage markers using Pearson’s product moment correlation coefficients (r). Statistical significance was set, a priori*¸* at *p* < 0.05. The statistical analysis was carried out using Statistica Version 13.2 software (StatSoft, France) and R (R Core Team (2018). R: A Language and environment for statistical computing. [Computer software]. Retrieved from https://cran.r-project.org/) using the *Car:Anova* package (Fox & Weisberg [45]; car: Companion to Applied Regression. [R package]. Retrieved from https://cran.r-project.org/package=car).

## Results

An a posteriori power calculation was computed using G*Power (Version 3.1, University of Düsseldorf, Germany) and the primary outcome parameter of this study MAV. As input parameters for G*power, we used the following variables from this study: effect size for MAV group by time interaction (η_p_^2^ = 0.61, equals Cohen’s f = 1.25), alpha error = 0.05, N = 29 total sample size, three groups and two tests. The post hoc power analysis showed an actual power of 0.99.

No testing or training related injuries occurred over the study period, and the attendance rates were 100% for the two exercise groups (e.g., EG_100_ and EG_110_). Pre and post test results are outlined in Tables 3 and 4. No significant between-group baseline differences were found for any of the analyzed parameters.

**Table 3:** Mean (± SD) values for muscle-damage markers measured during 30 s intermittent exercise before and after HIIT

Variables	Group	Pre	Post	*p* (eta-squared [η_p_^2^])		
				Main effect of group	Main effect of time	Group × time interaction
ALAT (UI/L)	EG_100_	20.4 ± 3.4	18.6 ± 3.4 a	0.379 (0.08)	< 0.001 (0.44)	< 0.001 (0.57)
	EG_110_	20.0 ± 3.4	17.0 ± 3.5 ac			
	CG	20.4 ± 3.6	20.7 ± 3.5			
ASAT (UI/L)	EG_100_	21.1 ± 4.4	19.2 ± 3.5 a	0.851 (0.02)	< 0.001 (0.56)	0.006 (0.29)
	EG_110_	20.3 ± 4.8	17.4 ± 3.7 ac			
	CG	20.2 ± 3.7	19.6 ± 2.9			
CRP (mg/L)	EG_100_	4.0 ± 0.8	3.5 ± 0.5 a	0.395 (0.08)	< 0.001 (0.41)	0.019 (0.17)
	EG_110_	3.5 ± 1.1	3.2 ± 0.8 a			
	CG	3.8 ± 0.7	3.7 ± 0.7			
CK (Ul/L)	EG_100_	201.9 ± 65.7	179.5 ± 58.6	0.831 (0.02)	< 0.001 (0.48)	0.008 (0.28)
	EG_110_	197.0 ± 43.7	166.0 ± 40.8 a			
	CG	205.3 ± 68.7	192.9 ± 51.8			
LDH (Ul/L)	EG_100_	199.7 ± 26.0	173.5 ± 28.3 a	0.843 (0.07)	0.004 (0.29)	0.012 (0.37)
	EG_110_	208.8 ± 67.8	167.0 ± 47.8 ac			
	CG	194.8 ± 24.5	192.8 ± 18.6			

EG_100_, trained group with 100% MAV (n = 10); EG_110_, trained group with 110% MAV (n = 10); CG, control groups without HIIT (n = 9); HIIT, High intensity interval training program; pre-test, before HIIT; post-test, after HIIT; ALAT, alanine aminotransferase; ASAT, aspartate aminotransferase; CK, creatine kinase; CRP, C-reactive protein, LDH, lactate dehydrogenase

a: significant difference between before and after HIIT

c: significant difference between EG_100_ and EG_110_

**Table 4:** Mean (± SD) values for hematological parameters measured during 30 s intermittent exercise pre, post HIIT

Variables	Group	Pre	Post	*p* (eta-squared [η_p_^2^])		
				Main effect of group	Main effect of time	Group x time interaction
WBC (10^3^ /μl)	EG_100_	7.1 ± 1.1	6.7 ± 1.1 a	0.646 (0.04)	< 0.001 (0.43)	0.013 (0.26)
	EG_110_	7.1 ± 1.1	6.6 ± 1.2 a			
	CG	7.3 ± 0.8	7.3 ± 0.8			
NE (10^3^ /μl)	EG_100_	4.0 ± 0.6	3.7 ± 0.5 a	0.632 (0.04)	< 0.001 (0.41)	0.031 (0.22)
	EG_110_	4.1 ± 0.9	3.7 ± 0.8 a			
	CG	3.9 ± 0.6	4.0 ± 0.6			
LY (10^3^ /μl)	EG_100_	2.6 ± 0.8	2.5 ± 0.8	0.919 (0.02)	< 0.001 (0.33)	0.005 (0.19)
	EG_110_	2.7 ± 0.8	2.1 ± 0.2 ac			
	CG	2.5 ± 0.6	2.5 ± 0.7			
MO (10^3^ /μl)	EG_100_	0.6 ± 0.2	0.5 ± 0.1	0.886 (0.02)	0.051 (0.10)	0.351 (0.08)
	EG_110_	0.6 ± 0.1	0.5 ± 0.1			
	CG	0.6 ± 0.1	0.5 ± 0.1			

EG_100_, trained group with 100% MAV (n = 10); EG_110_, trained group with 110% MAV (n = 10); CG, control groups without HIIT (n = 9); HIIT, High intensity interval training program. pre-test, before HIIT; post-test, after HIIT; WBC, white blood cells; NE, neutrophils; LY, lymphocytes; MO, monocytes

a: significant difference between before and after HIIT

c: significant difference between EG_100_ and EG_110_

### Maximal aerobic velocity (MAV)

A significant main effect of time was observed for MAV performances (F = 84.39; *p* < 0.001; η_p_^2^ = 0.71; power = 0.99). A significant group × time interaction was found for MAV (F = 18.26; *p* < 0.001; η_p_^2^ = 0.61; power = 0.99). Post-hoc tests revealed significant (*p* < 0.001) pre-to-post improvements for MAV performances in EG_100_ (η_p_^2^ = 0.08, + 5.8%) and EG_110_ (η_p_^2^ = 0.10, + 8.9%) compared to CG. Post hoc tests revealed also higher MAV performances (*p* = 0.01; η_p_^2^ = 0.05) in EG_110_ compared to EG_100_ after HIIT. A pilot study was recorded to assess the reliability and the sensitivity of this test using 29 participants and showed 0.94 ICC and 95% CI (0.88–0.98).

### Muscle damage markers

Significant main effects of time were observed for ALAT (F = 27.21; *p* < 0.001; η_p_^2^ = 0.68; power = 0.99), ASAT (F = 14.49; *p* < 0.001; η_p_^2^ = 0.66; power = 0.95), CRP (F = 6.05; *p* < 0.001; η_p_^2^ = 0.41; power = 0.66), CK (F = 18.31; *p* < 0.001; η_p_^2^ = 0.82; power = 0.98), and LDH (F = 13.45; *p* < 0.001; η_p_^2^ = 0.61; power = 0.95). Significant group × time interactions were identified for ALAT (F = 15.54; *p* = 0.007; η_p_^2^ = 0.16; power = 0.99), ASAT (F = 1.92; *p* = 0.004; η_p_^2^ = 0.17; power = 0.88), CRP (F = 8.50; *p* = 0.009; η_p_^2^ = 0.02; power = 6.21), CK (F = 9.28; *p* = 0.003; η_p_^2^ = 0.08; power = 0.84), and LDH (F = 5.97; *p* = 0.006; η_p_^2^ = 0.33; power = 0.93). Post hoc tests revealed significant pre-to post decreases (*p* < 0.001) in both EG_100_ and EG_110_ for ALAT (η_p_^2^ = 0.06; η_p_^2^ = 0.16, respectively) and ASAT (η_p_^2^ = 0.05; η_p_^2^ = 0.10, respectively). Post hoc tests revealed that ALAT (*p* = 0.04; η_p_^2^ = 0.05) and ASAT (*p* = 0.02; η_p_^2^ = 0.06) were significantly lower in EG_110_ (ALAT:—3.0 ± 1.6; ASAT: − 2.9 ± 3.7) compared to EG_100_ (ALAT: − 1.8 ± 0.9; ASAT: − 1.9 ± 1.7) after HIIT. Post hoc tests revealed significant pre-to post decreases for CRP in EG_100_ (*p* = 0.03; η_p_^2^ = 0.12; − 12.5%) and EG_110_ (*p* = 0.02; η_p_^2^ = 0.02; − 8.6%) and for LDH in EG_100_ (*p* = 0.005; η_p_^2^ = 0.19; − 13.1%) and EG_110_ (*p* < 0.001; η_p_^2^ = 0.11; − 20%). However, CK decreased only for EG_110_ (*p* = 0.01; η_p_^2^ = 0.12, − 15.7%), after HIIT.

A pilot study was recorded to assess the reliability and the sensitivity of this parameters using 29 participants and showed 0.91 ICC and 95% confidence interval (CI) of 0.83–0.95 for ALAT, 0.89 ICC and 95% CI of 0.72–0.96 for ASAT, 0.93 ICC and 95% CI of 0.75–0.98 for CRP, 0.95 ICC and 95% CI of 0.89–0.96 for CK and 0.95 ICC and 95% CI of 0.84–0.98 for LDH.

### Contrast analysis (muscle damage)

Contrast analysis indicated that adherence to any HIIT condition (EG_100_ and EG_110_) significantly reduced ALAT (Est:2.62, SE:0.51, t(28) = 5.07, *p* < 0.001) and LDH (Est:32, SE:9.09, t(28) = 3.51, *p* = 0.001) vs. the control condition. However, contrast analysis highlighted no significant changes for ASAT (Est:1.73, SE:1.06, t(28) = 1.63, *p* = 0.11), CRP (Est:0.28, SE:0.26, t(28) = 1.08, *p* = 0.28), and CPK (Est:14.25, SE:14.82, t(28) = 0.96, *p* = 0.34), for HIIT conditions vs. control.

### Hematological parameters

Significant main effects of time were found for WBC (F = 14.92; *p* < 0.001; η_p_^2^ = 0.82; power = 0.96), NE (F = 24.72; *p* < 0.001; η_p_^2^ = 0.59; power = 0.99), LY (F = 12.41; *p* < 0.001; η_p_^2^ = 0.73; power = 0.93), and MO (F = 7.22; *p* < 0.001; η_p_^2^ = 0.62; power = 0.74). Significant group × time effects were observed for WBC (F = 0.81; *p* = 0.022; η_p_^2^ = 0.14; power = 0.99), NE (F = 5.49; *p* = 0.015; η_p_^2^ = 0.15; power = 0.71), LY (F = 8.97; *p* = 0.002; η_p_^2^ = 0.17; power = 0.83), and MO (F = 5.76; *p* = 0.004; η_p_^2^ = 0.10; power = 0.63). Post hoc tests revealed significant pre-to post decreases in both EG_100_ and EG_110_ for WBC (*p* = 0.013, *p* = 0.002, respectively; η_p_^2^ = 0.03, η_p_^2^ = 0.04, respectively; − 5.6%, − 7.0%, respectively), and for NE (*p* < 0.001; η_p_^2^ = 0.07, η_p_^2^ = 0.05, respectively; − 7.5%, − 9.8%, respectively). However, post hoc tests revealed a significant decrease in LY (*p* = 0.02; η_p_^2^ = 0.21, − 22.2%) only in EG_110_ after HIIT.

A pilot study was conducted to assess the reliability and the sensitivity of this parameters using 29 participants, and showed an ICC of 0.97 and 95% CI of 0.91—0.99 for WBC, 0.89 ICC and 95% CI of 0.78—0.94 for NE, 0.87 ICC and 95% CI of 0.69—0.88 for LY and 0.89 ICC and 95% CI of 0.78—0.93 for MO.

### Contrast analysis (hematology)

Contrast analysis indicated that any HIIT condition (EG_100_ and EG_110_) significantly reduced WBC (Est:0.45, SE:0.11, t(28) = 4.29, *p* = 0.002) and NE (Est:0.33, SE:0.07, t(28) = 4.68, *p* < 0.001) vs. the control condition. However, contrast analysis highlighted no significant changes for LY (Est:0.32, SE:0.16, t(28) = 2.03, *p* = 0.06) and MO (Est:0.06, SE:0.06, t(28) = 1.12, *p* = 0.27), for HIIT conditions vs. control.

### Relationships between deltas in MAV with deltas in hematological parameters and muscle-damage markers

Table 5 illustrates that pre-post changes in MAV correlated with pre-post changes in hematological and muscle-damage markers in the range of 0.40 to 0.79. Significant correlations were found between MAV and ALAT (r = 0.66, *p* < 0.05), MAV and CK (r = 0.67, *p* < 0.05), MAV and LDH (r = 0.74, *p* < 0.05), MAV and LY (r = 0.79, *p* < 0.01) for EG_100_. However, there were no significant correlations between MAV and ASAT, CRP and WBC for EG_100_.

**Table 5:** Correlations between percentage changes of maximal aerobic velocity (MAV) and deltas in hematological and muscle-damage markers

	% Change		Pearson's r (*p* values)	
	EG_100_	EG_110_	EG_100_	EG_110_
MAV	+ 5.9	+ 8.9%		
ALAT (UI/L)	− 9.0%	− 15.2%	0.66 (0.0438)*	0.64 (0.041)*
ASAT (UI/L)	− 8.3%	− 12.4%	0.54 (0.107)	0.39 (0.265)
CRP (mg/L)	− 11.0%	− 4.8%	0.40 (0.252)	0.07 (0.487)
CK (Ul/L)	− 10.7%	− 14.6%	0.67 (0.031)*	068 (0.030)*
LDH (Ul/L)	− 13.4%	− 18.5%	0.74 (0.014)*	0.71 (0.021)*
WBC (10^3^ /μl)	− 5.7%	− 7.5%	0.58 (0.078)	0.55 (0.099)
NE (10^3^ /μl)	− 7.1%	− 8.2%	0.61 (0.061)	0.66 (0.037)*
LY (10^3^ /μl)	− 5.1%	− 19.2%	0.79 (0.006)**	0.72 (0.018)**
MO (10^3^ /μl)	− 12.6%	− 10.4%	0.42 (0.226)	0.22 (0.541)

EG_100_, trained group with 100% MAV (n = 10); EG_110_, trained group with 110% MAV (n = 10); MAV, maximal aerobic velocity; ALAT, alanine aminotransferase; ASAT, aspartate aminotransferase; CK, creatine kinase; CRP, C-reactive protein; LDH, lactate dehydrogenase.WBC, white blood cells; NE, neutrophils; LY, lymphocytes; MO, monocytes. *Significant correlation, *p<0.05, **p<0.01

For EG_110_, significant correlations were found between MAV and ALAT (r = 0.64, *p* < 0.05), MAV and CK (r = 0.86, *p* < 0.05), MAV and LDH (r = 0.071, *p* < 0.05), MAV and LY (r = 0.72,* p* < 0.01) and MAV and NE (r = 0.66; *p* < 0.05). However, there were no significant correlations between MAV and ASAT, CRP and WBC for EG_110._

## Discussion

This study compared the effects of eight weeks of HIIT, at 100% MAV vs. 110% MAV, on performance development (i.e., MAV), muscle damage markers, and hematological parameters in young active men. The main findings of this study were that HIIT at 110% MAV induced higher MAV values compared to 100% MAV. Indeed, HIIT at 110% MAV induced lower ASAT, ALAT, LDH, NE, and LY concentrations than exercising at 100% MAV. There were significant correlations (*p* < 0.05) between MAV and ASAT, ALAT, LDH, NE, and LY in EG_100_ and EG_110_. However, no statistically significant effect of any exercise modality was observed for MO, WBC, CRP, and CK.

### Effects of increasing intensity on performance development

Our results showed a significant improvement in aerobic performances after HIIT in both trained groups compared to pre-intervention. Our findings agree with previous results reported in the literature. Indeed, several studies reported significant increases in V̇O_2max_ and MAV after 4 to 7 weeks of a HIIT program [46, 47]. In addition, in our study, MAV performances recorded after HIIT were significantly better in EG_110_ compared to EG_100_. These differences seem to be dependent on the intensity of exercise, since EG_100_ presented smaller improvement in MAV performance compared to EG_110_ [39], whilst both HIIT conditions yielded significant improvements vs. the control group.

### Muscular damages markers variations in response to HIIT

After HIIT training, we found significantly decreased responses compared to pre-intervention in ALAT, ASAT, CRP, and LDH, in both EG_100_ and EG_110_ groups. Furthermore, after HIIT, ALAT and ASAT concentrations were significantly lower in EG_110_ compared to EG_100_. Furthermore, specific contrast analysis indicated that adherence to any HIIT condition (EG_100_ and EG_110_) significantly reduced ALAT and LDH compared to the control condition. This study showed significant positive correlations between pre-post changes in MAV with deltas in ALAT for EG_100_ (r = 0.66; *p* < 0.05) and EG_110_ (r = 0.64; *p* < 0.05). These findings suggest that high intensity exercise has an impact on selected biochemical markers of muscular damage.

How training programs reduce inflammation and CRP levels is not well defined [48]. However, exercise training related reductions in fat mass have been associated with a larger reduction in CRP [34]. Musa et al. [33] showed significant decreases in high-density lipoprotein (HDL) cholesterol and triglycerides levels after 8 weeks of HIIT, whilst Kessler et al. [49], in their literature review, also reported that HIIT improved HDL cholesterol after a minimum of 8 weeks of HIIT. Physical activity is related to several factors that are independently correlated with lower CRP levels [50]. Moreover, Mauger et al. [51] noted that CRP production was significantly positively correlated with IL-6, and inversely correlated with HDL-C levels. Indeed, CRP metabolism has been linked to liver fat deposition, and therefore altered uptake of triglyceride-rich lipoprotein would be predicted to affect CRP metabolism [52]. Thus, the decrease of CRP observed in the present study could be explained by cholesterol and triglycerides’ concentrations.

After the HIIT program, CK levels were significantly lower in the EG_110_ group compared to before training. Our results are concordant with those of Baird et al. [53], who showed that, although the mechanism(s) by which CK is cleared from the blood has not been fully elucidated, it is likely that the observed serum CK levels reflect complex interactions associated with energy status and scale of muscle disturbance [53]. In agreement, Brewster et al. [54] showed that higher levels of tissue CK activity may increase the cellular energy and improve muscular contraction. However, it is less clear why physical exercise should result in release of CK into the blood [53]. Moreover, according to Baird et al. [53], when total work time is equalized by inversely varying intensity and duration, the greatest rise in CK levels occurs in the highest-intensity (80% MAV) exercise with the shortest duration (170 repetitions), compared to the session with a longer duration (524 repetitions) and lower intensity exercise (30% MAV). Indeed, these results support the possibility of higher CK levels with increasing exercise intensity.

Main et al. [55] showed that increases in muscle damage could explain the decrease in muscle performance after 8 weeks of endurance training program. Thus, the decrease of muscle damage we observed (i.e., CK) after HIIT, in both EG_100_ and EG_110,_ could be partly explanatory for the increasing performance. Furthermore, in our study, after HIIT, MAV values were significantly higher in EG_110_ than EG_100_. In this study, we observed a significant positive correlation between deltas in MAV with pre-post changes in CK (r = 0.67; r = 0.86; respectively; all *p* < 0.05) for EG_100_ and EG_110_.

Therefore, HIIT seems to improve aerobic performances, by increasing intermittent exercise intensity, without a notable difference in muscular damage markers level. According to Morgan [56], the inflammatory response during exercise is related to the muscular metabolism and the degree of muscular lesion development. Morgan [56] suggested that the subsequent muscular adaptation process involved an increase in the number of sarcomeres in series of muscle fibers after a training program, based on specific repeated running training. Thus, after a HIIT program, compared to before, the decrease of muscular damage markers observed in both groups in the present study could be manifest through muscular adaptations processes. Accordingly, we speculate that the 30 s intermittent exercise with 110% MAV probably lead to non-uniform sarcoma elongation, that could explain muscles adaptations compared to 100% MAV [56]; of course, the veracity of this claim must be addressed in further mechanistic studies.

Our study showed that LDH concentrations were significantly lower in EG_110_ compared to EG_100_ after the HIIT program. The LDH decrease in EG_110_ after HIIT could be mainly attributed to aerobic pathway intervention. Indeed, according to the duration, the human body uses increasingly aerobic pathway to produce a sufficient quantity of ATP for exercise [57]. This action promotes the decrease of LDH activity [58, 59], and can explain the diminution of LDH found in our study after HIIT using 110% MAV. Previous studies have reported a significant increase of LDH in response to acute exercise, such as weightlifting, high intensity exercise, and after maximal graded tests [60, 61]. This increase could be explained by the increase of V̇O_2_ during exercise_,_ which causes oxidative stress and muscle damage [62]. This study showed significant positive correlations for between MAV and LDH (r = 0.74, r = 0.071, respectively; *p* < 0.05) for EG_100_ and EG_110_.

### Hematological parameters variations in response to HIIT

Our study showed that WBC and NE decreased significantly in both EG_100_ and EG_110_ after HIIT compared to pre-intervention. However, LY decreased significantly only in the EG_110_ group after HIIT compared to before. Furthermore, specific contrast analysis indicated that adherence to any HIIT condition (EG_100_ and EG_110_) significantly reduced WBC and NE compared to the control condition. There was a significant positive correlation between MAV and and LY (r = 0.79, r = 0.72, respectively; *p* < 0.01) for EG_100_ and EG_110_. The decreases in WBC, NE and LY previously defined after HIIT could be reflective of the "open window" concept [63]. Indeed, in monitoring the changes in humoral and cellular immune parameters following endurance exercise, it has been proposed that this type of exercise can induce a suppression of some immune variables which may make the individuals more susceptible to pathogens [64]. Our results were in accordance with those of Morgado et al. [7], who showed that the cumulative effects of training loads induced an overall reduction of the ability of the immune system to respond after a training program. Kakanis et al. [65] found that intermittent exercise training (at 90% of the subject’s second ventilatory threshold) provoked a temporary reduction in immune function (i.e., respiratory burst response, proliferation). However, Nieman [66] found that moderate intensity (< 3 moderate-to vigorous aerobic session of > 20 min) training program, over 15 weeks (5 sessions / week), induced an increase (+ 20%) in immunoglobulin and a diminution in LY. Likewise, many studies do not indicate changes in immune function past 2 h after the completion of exercise.

### Strengths and limitations of the study

The present study represents a novel addition to the literature; indeed, to our knowledge, there are no published data concerning increasing the exercise intensity of intermittent exercise and the resultant muscle damage and hematological responses. We hypothesized that increasing intensity training could lead to better performance development (MAV) in order to reduce muscular damage markers after short and intense intermittent exercise, and that any HIIT would yield greater changes than the control group. Accordingly, these hypotheses were robustly, statistically, addressed using ANOVA and post-hoc testing, including specific contrast analysis. As to this study’s limitations, we acknowledge that a greater sample size would have been preferable, and further work that is appropriately powered is now required. However, we assert that this work represents the first of its’ type, and will provide a platform for further research. While our study focused on measures of aerobic performance, biochemical and hematological parameters to examine the muscular damages, a comparison of muscle soreness and muscular strength could further strengthen and explain our findings.

### Practical applications

When coaches prescribe exercise programs, two essential aims are sought; improve performance and avoid injuries. To that end, we suggest the HIIT program based on IE at 110% MAV appears feasible, because it does not induce additional muscle damage compared to the intervention group that performed at 100% MAV. However, based on contrast analysis, it is evident that participating to either HIIT protocols outlined in the present study would elicit significant muscular and hematological improvements.

## Conclusions

In conclusion, 8-weeks of HIIT, at both 100% and 110% MAV, had significant positive effects on damage and hematological measures, vs. control. Moreover, training HIIT at a 110% MAV intensity was more efficient for improving MAV with + 9.07 ± 3.38% and reducing damage biomarkers (ALAT, ASAT, CK and LDH), vs. 100% MAV, in physically active young men.

## Data Availability

The datasets generated during and analyzed during the current study are not publicly available due to confidential information about the participants but are available from the corresponding author on reasonable request.

## Abbreviations

HIIT: High-intensity interval training programs
MAV: Maximal aerobic velocity
V̇O_2_: Oxygen consumption
ASAT: Aspartate aminotransferase
ALAT: Alanine aminotransferase
CRP: C reactive protein
CK: Creatine kinase
WBC: White blood cells
MO: Monocytes
LY: Lymphocytes
NE: Neutrophil
LDH: Lactate dehydrogenase
ANOVA: 2-Way analysis of variance
ES: Effect size
